# Factors Involved in Tuberculosis Recurrence in a Low-incidence Setting; Reactivation Predominates Over Reinfection in a 30-year Surveillance Study

**DOI:** 10.1093/ofid/ofag079

**Published:** 2026-02-18

**Authors:** Xunxiao Lin, Daniel Ibarz, Alberto Cebollada, Carlos Martín, María-José Iglesias, Sofía Samper

**Affiliations:** Grupo de Genética de Micobacterias, Instituto de Investigación Sanitaria (IIS) Aragón, Zaragoza, Spain; Facultad de Medicina, Universidad de Zaragoza, Zaragoza, Spain; Grupo de Genética de Micobacterias, Instituto de Investigación Sanitaria (IIS) Aragón, Zaragoza, Spain; Facultad de Medicina, Universidad de Zaragoza, Zaragoza, Spain; Unidad de Biocomputación, Instituto Aragonés de Ciencias de la Salud, Zaragoza, Spain; Grupo de Genética de Micobacterias, Instituto de Investigación Sanitaria (IIS) Aragón, Zaragoza, Spain; Facultad de Medicina, Universidad de Zaragoza, Zaragoza, Spain; Centro de Investigación Biomédica en Red (CIBER) de Enfermedades Respiratorias, Madrid, Spain; Grupo de Genética de Micobacterias, Instituto de Investigación Sanitaria (IIS) Aragón, Zaragoza, Spain; Facultad de Medicina, Universidad de Zaragoza, Zaragoza, Spain; Centro de Investigación Biomédica en Red (CIBER) de Enfermedades Respiratorias, Madrid, Spain; Grupo de Genética de Micobacterias, Instituto de Investigación Sanitaria (IIS) Aragón, Zaragoza, Spain; Facultad de Medicina, Universidad de Zaragoza, Zaragoza, Spain; Centro de Investigación Biomédica en Red (CIBER) de Enfermedades Respiratorias, Madrid, Spain; Instituto Aragonés de Ciencias de la Salud, Zaragoza, Spain

**Keywords:** *Mycobacterium tuberculosis*, Relapse, Reinfection, Reactivation, multimorbidity

## Abstract

**Background:**

Tuberculosis (TB) recurrence remains a significant public health concern, even in regions with low incidence. Recurrent TB may result from endogenous reactivation of a previous infection or from exogenous reinfection with a new strain. Distinguishing between these mechanisms is crucial for understanding TB dynamics and optimizing control strategies. This study aims to determine the frequency of TB recurrence in Aragón, Spain, a region with low TB incidence, and to identify factors associated with reactivation and reinfection over a 30-year period.

**Methods:**

A retrospective, descriptive study including all genotyped *Mycobacterium tuberculosis* isolates from 1993 to 2022 was conducted in Aragón. IS*6110*-RFLP was the method used to genotype strains. Recurrences were classified as reactivation or reinfection based on molecular profiles. Clinical and epidemiological data were retrieved from medical records. Appropriate statistical tests were applied to compare groups.

**Results:**

Among 3510 genotyped TB cases, 81 (2.30%) were recurrent: 68 reactivations (1.93%) and 15 reinfections (0.42%). Reinfection was significantly associated with change of residence, HIV infection, cancer diagnosis in the second episode, and multimorbidity. Time to recurrence was significantly longer in reinfections (median 7.0 years) compared to reactivations (2.0 years). Most isolates belonged to Lineage 4 , and reinfection strains were more often linked to clustered strains circulating in the community.

**Conclusions:**

In this low-incidence setting, TB recurrence is rare and mainly the result of reactivation. Reinfections, though less frequent, are linked to mobility, HIV co-infection, neoplasm, and compromised health status. These findings underscore the importance of long-term molecular surveillance and targeted follow-up for high-risk patients.

## Teaser

Tuberculosis recurrence in Aragón (1993–2022) was rare (2.30%). Reactivation predominated over reinfection, which was associated with mobility, HIV, cancer status, and multimorbidity. Molecular surveillance and targeted follow-up are essential to address recurrence dynamics in low-incidence settings.

Tuberculosis (TB) once again became the leading cause of death from a single infectious agent globally, surpassing COVID-19, after a 3-year interval [1]. The World Health Organization (WHO) estimates that 10.8 million individuals developed TB in 2023, with 1.25 million deaths worldwide. Transmission occurs primarily through airborne inhalation of droplets containing *Mycobacterium tuberculosis* bacilli. Although many infections resolve spontaneously, approximately 5%–10% of individuals with latent TB will experience reactivation and develop active disease [2].

According to WHO definitions, TB recurrence or relapse refers to patients who were previously treated for TB, declared cured or treatment-completed at the end of their last treatment cycle, and are diagnosed with a new episode of TB, either a reactivation or an exogenous reinfection [3]. Prior TB is 1 of the strongest predictors of future disease, with recurrence being up to 14 times more likely than a new primary infection [4]. Despite the availability of effective anti-TB treatment, therapeutic success remains suboptimal, with global success rates below 85% [5]. Recurrent TB is associated with worse outcomes, including higher mortality [6, 7], increased community transmission, and potential outbreak generation [8]. According to WHO, recurrences of TB account for approximately 7% of incident cases [5]. Recurrence rates vary geographically and are typically higher in high-incidence regions compared to low-incidence settings [9]. Identifying risk factors for recurrence is essential to guide public health interventions. Known risk factors include HIV infection, smoking, diabetes, residual pulmonary cavitation, poor adherence to treatment, and the specific strain of *M tuberculosis* involved [10–14].

Genotyping techniques are crucial for distinguishing between reactivation (suggesting treatment failure or latent persistence) and reinfection with a new strain (suggesting renewed exposure and transmission) [15], with critical implications for TB surveillance, outbreak control, and vaccine design. Recent conceptual advances have challenged the static view of TB latency. In a landmark review, Behr et al. propose a dynamic model of TB pathogenesis in which risk of progression and recurrence fluctuates over time, modulated by host, pathogen, and environmental factors [16]. Importantly, this model identifies the first 2 years after infection or treatment as the highest risk period, with later recurrences more likely reflecting reinfection. This nuanced framework underscores the importance of temporal and mechanistic distinctions in both clinical management and research. These insights are particularly relevant for TB vaccine development. Traditionally, some vaccine trials, such as the H56:IC31 trial evaluated by Borges et al., used TB recurrence as a primary endpoint to shorten follow-up and reduce sample sizes [17]. However, they highlight, recurrence-based endpoints are limited by low event rates and mechanistic heterogeneity, especially in low-incidence settings. There is now broad consensus that prevention of disease should be the preferred efficacy endpoint for future vaccine trials, particularly in infection-naïve populations. In this context, long-term surveillance studies that precisely differentiate reactivation and reinfection remain essential to inform trial design, risk stratification, and postlicensure monitoring.

TB is a notifiable disease in Spain, where patients receive free healthcare. Systematic genotyping of *M tuberculosis* isolates has been performed since 2004 in Aragón. This study represents a unique opportunity to analyze 3 decades of molecular and clinical data, to quantify the incidence of reactivation and reinfection, identifying the associated risk factors in a representative low-incidence population.

## METHODS

### Study Design and Setting

This retrospective study was conducted in Aragón, a demographically stable region in northeastern Spain, with a population of 1.35 million inhabitants. In 2023, the TB notification rate was 7.31 cases per 100 000 population [18], below the national average for that year (8.2 per 100 000). In Aragón, TB care is organized within the public health system, with diagnosis, treatment, and follow-up coordinated through specialized hospital services and primary care in collaboration with the regional TB control program. Microbiological diagnosis is routinely performed using sputum or other clinical samples, which are referred to the regional reference laboratory for mycobacteriology for culture confirmation and drug susceptibility testing.

All genotyped *M tuberculosis* strains from TB cases diagnosed between 2004 and 2022 were included (2557 cases). Given the mandatory notification of TB and the centralized organization of microbiological diagnosis, this study captured nearly all culture-confirmed TB cases diagnosed in Aragón during the study period. IS*6110-*RFLP was considered the gold standard until the introduction of whole-genome sequencing (WGS), and remains a valid method for distinguishing between reactivation and reinfection [15]. Historical genotypes of previous population-based studies in Zaragoza province (including 561 cases in 1993–1995 and 392 in 2001–2004), were available [19]. This collection of genotypes, referred to as ARAMOL, is integrated into the Bionumerics v7.6 software (Applied Maths, Kortrijk, Belgium), which enables comparison of all strains and identified patterns over time. Case definitions followed those established in the European Union (EU) case definition protocol as published in the Official Journal of the European Union (Commission Implementing Decision [EU] 2018/945) [20] and in the National Epidemiological Surveillance Network protocol (Spain) [21].

We defined recurrent TB cases as those TB recurrence patients either a reactivation or an exogenous reinfection with more than 1 positive culture for *M tuberculosis*, with a time interval between cultures greater than 180 days. This threshold aligns with the duration of a standard treatment regimen in Spain for pulmonary TB cases without suspicion of drug resistance: an initial 2-month intensive phase with 4 drugs (isoniazid, rifampicin, ethambutol, and pyrazinamide) followed by a continuation phase of 4 months with 2 drugs (isoniazid and rifampicin). Cases with multiple positive cultures within 180 days were excluded because they were considered part of the same episode. Reactivation was defined as a recurrence involving the same strain (identical IS*6110*-RFLP pattern, followed by genome sequence if available DNA). Reinfection was defined as recurrence with a different strain IS*6110*-RFLP pattern. Genomes sequences are available in GenBank (accession SAMN26037035–SAMN26037070; BioProject ID PRJNA808219) [15].

Clinical records of all included cases were reviewed to extract social class (Supplementary Table 1) [22], demographic and clinical and behavioral variables: age, sex, country of birth, comorbidities, immunosuppressive status, chest X-ray findings, treatment adherence, and outcome.

### Statistical Analysis

Categorical variables were analyzed using the chi-squared or Fisher exact test. For continuous variables, *t*-test and the Wilcoxon–Mann–Whitney test was used otherwise. The Shapiro–Wilk test was applied to assess the normality of numeric variables. A *P*-value < .05 was considered statistically significant.

## RESULTS

**Figure 1.**
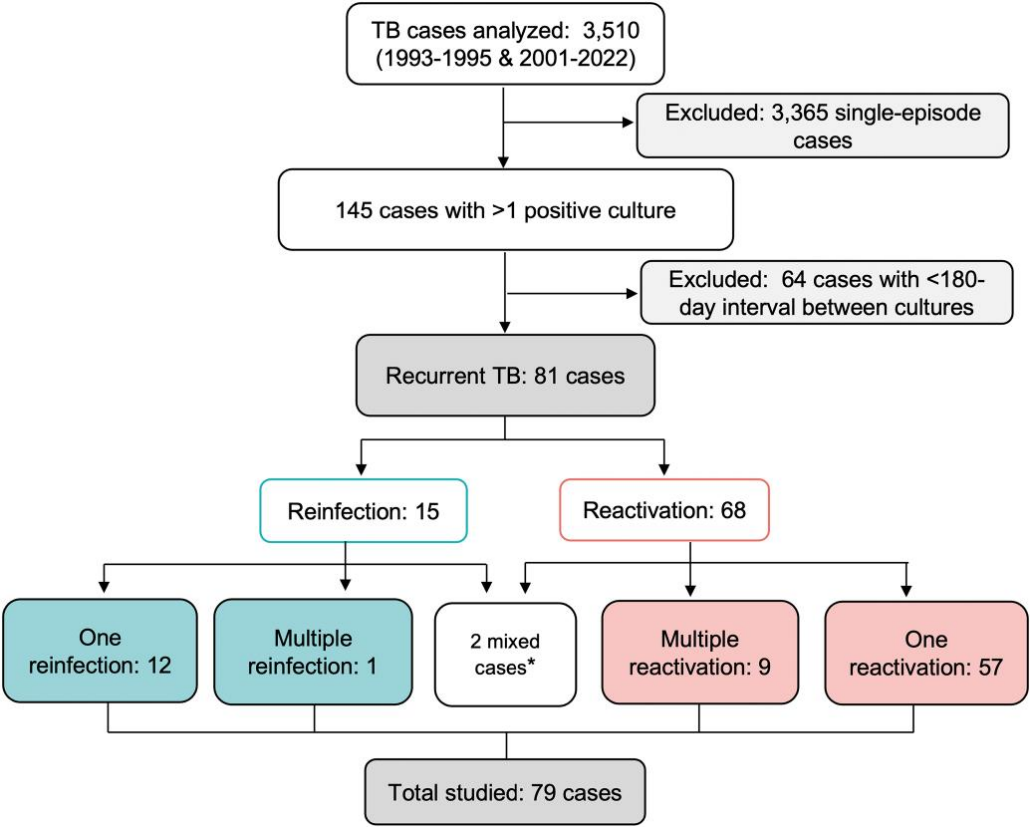
Flow chart of tuberculosis cases included and excluded from the study. *Two mixed cases were excluded from the subsequent analysis.

In Aragón, totalling 3686 genotyped *M tuberculosis* isolates from 3510 patients (Figure 1). Of these, 3365 cases were considered nonrecurrent. A total of 145 cases presented multiple isolates; 64 were excluded because of short intervals (<180 days), leaving 81 as recurrent TB. Among these, 15 showed exogenous reinfections: 12 with a single reinfection, 1 presenting 2 reinfections, and finally, 2 in which both forms of recurrence were observed. As for endogenous reactivations, 68 cases had at least 1 endogenous reactivation: 57 with a single reactivation, 9 with multiple reactivations, and 2 cases in which both reinfection and reactivation were detected (as mentioned previously). The percentage of recurrence was 2.30%, 1.93% from reactivation and 0.42% from reinfection. Case c53 showed a reactivation 5 years after the first episode followed by reinfection 5 years later, whereas Case c64 presented reinfection 10 years after the initial episode and a subsequent reactivation 7 years later. To facilitate the comparative study of exogenous reinfection versus endogenous reactivation, both cases were excluded from the analysis. Finally, 79 cases were included: 13 reinfection cases, 1 of them presented 2 reinfections, and 66 reactivation cases, of these, 57 experienced a single reactivation; 6 had 2 reactivations; 2 had 3 reactivations; and 1 case presented 5 reactivations during the study period.

### Sociodemographic and Clinical Characteristics

**Figure 2.**
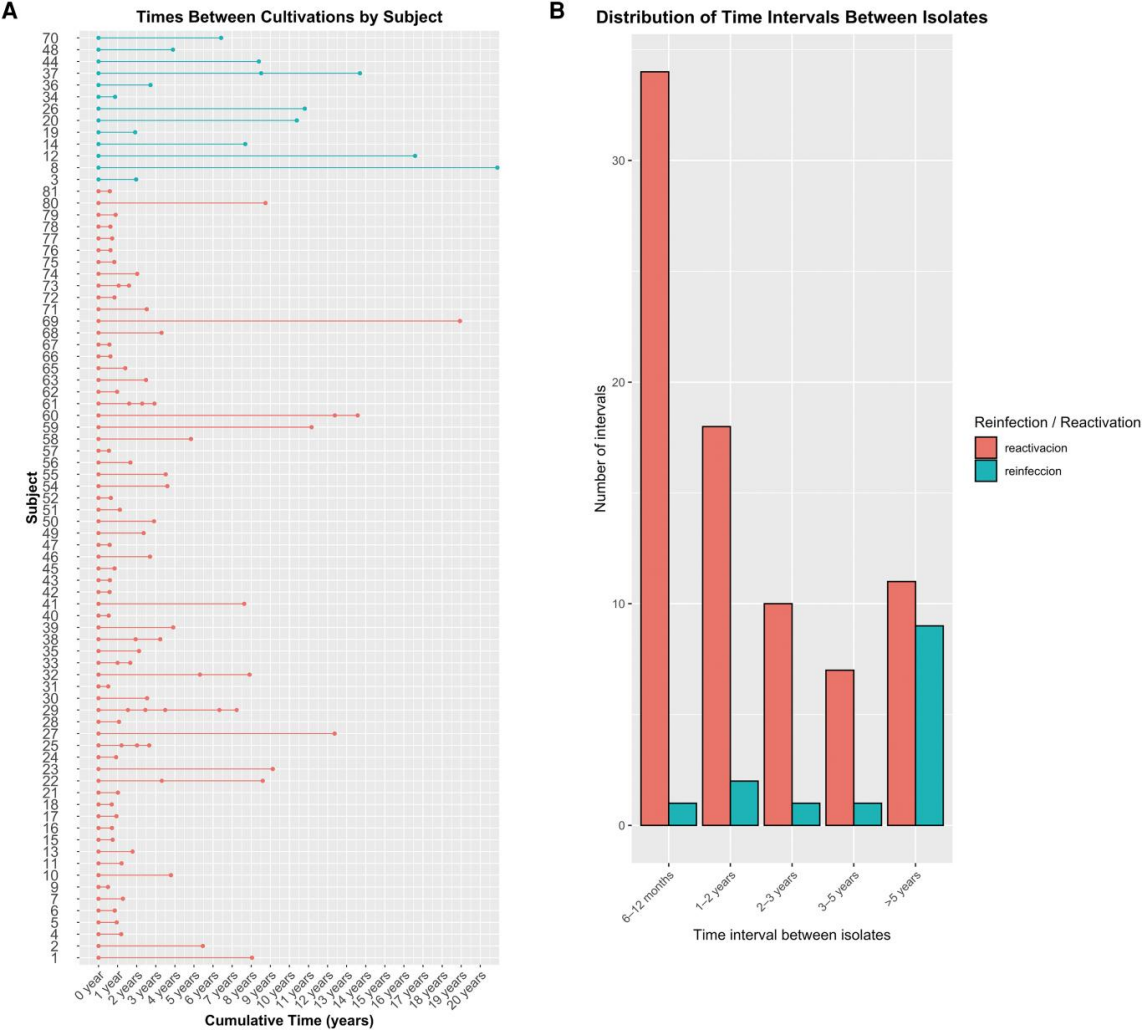
(A) Visualization of the time between recurrent infection episodes in the same patient. (B) Visualization of the distribution of time intervals between isolates. Blue line: cases of reinfections. Red line: cases of reactivations.

Recurrent tuberculosis (n = 79) mostly affected males (79.7%), with median ages at first and last episodes of 39 and 43 years (Table 1). Most (65.0%) lived in Zaragoza City, 33.3% were foreign-born, 56.9% came from families facing social challenges, and 88.0% belonged to manual or unskilled workers (social classes IV/V). Hospitalization for diagnosis or treatment occurred in 88.6% of the cases. No significant sociodemographic differences were noted between reactivation and reinfection, except in residence change: 66.7% of reinfection cases moved, versus 15.4% of reactivation (*P* = .002). At least 1 comorbidity was present in 51.9% of total cases, rising to 84.6% in the reinfections (*P* = .010). HIV co-infection was higher in reinfection cases (58.3% vs 20.3%; *P* = .011). No significant differences were observed in the prevalence of diabetes mellitus, silicosis, chronic renal failure, AIDS, or neoplasm. The occurrence of neoplasm in reinfection cases after the first episode of TB was significant (*P* = .016). To assess the potential impact of multimorbidity on recurrence, we calculated the total number of comorbidities per patient, considering diabetes, silicosis, chronic renal failure, AIDS, neoplasm, and other causes of immunosuppression (either treatment-related or from other chronic conditions). Patients with reinfection had a significantly higher number of comorbidities than those with reactivation (*P* = .004); Specially, 54.5% of reactivation patients had none, whereas only 15.4% of reinfection patients did. Pulmonary radiology showed no differences. Most reactivation patients (63.6%) completed treatment in at least 1 episode of disease, versus 100% in reinfection, with slight differences in lost-to-follow-up rates in any episode (83.3% in reactivation vs 80.0% in reinfection). Mortality was 19.6% in reactivation and 28.6% in reinfection. Isoniazid resistance was found in 8 reactivation patients in the first episode and 7 during reactivation, with some resistance to rifampicin and streptomycin developing later. All strains in reinfection remained susceptible (Table 2). The median time to recurrence was 2.00 (1.00, 3.00) years for reactivation and 7.00 (3.00, 10.00) years for reinfection (*P* < .001) (Figure 2).

**Table 1.** Demographic, Socioeconomic, Clinical, and Behavioral Characteristics of Tuberculosis Recurrence Cases Enrolled in This Study

		Overall	Reactivation	Reinfection	*P* Value
	N	N = 79	N = 66	N = 13	
Sex	79				.4
Man		63/79 (79.7%)	54/66 (81.8%)	9/13 (69.2%)	
Woman		16/79 (20.3%)	12/66 (18.2%)	4/13 (30.8%)	
Age					
First infection Median (Q1, Q3)	78	39.00 (31.00, 49.00)	39.00 (31.00, 50.00)	36.00 (34.00, 39.00)	.5
Last infection Median (Q1, Q3)	77	43.00 (36.00, 52.00)	42.00 (35.00, 53.00)	46.50 (38.50, 49.00)	.4
Foreign-born	78	26/78 (33.3%)	22/66 (33.3%)	4/12 (33.3%)	>.9
Change of health district	74	16/74 (21.6%)	10/65 (15.4%)	6/9 (66.7%)	**.002**
Families facing social challenges	51	29/51 (56.9%)	22/41 (53.7%)	7/10 (70.0%)	.5
Social class^a^	25				.9
I	…	1/25 (4.0%)	1/22 (4.5%)	0/3 (0.0%)	
II	…	1/25 (4.0%)	1/22 (4.5%)	0/3 (0.0%)	
III	…	1/25 (4.0%)	1/22 (4.5%)	0/3 (0.0%)	
IV	…	13/25 (52.0%)	12/22 (54.6%)	1/3 (33.3%)	
V	…	9/25 (36.0%)	7/22 (31.8%)	2/3 (66.7%)	
Hospital admission	79	70/79 (88.6%)	57/66 (86.4%)	13/13 (100.0%)	.3
Risk behaviours					
Tobacco use	63	52/63 (82.5%)	43/51 (84.3%)	9/12 (75.0%)	.4
Alcohol consumption	59	44/59 (74.6%)	35/48 (72.9%)	9/11 (81.8%)	.7
Injecting drug users	63	9/63 (14.3%)	6/50 (12.0%)	3/13 (23.1%)	.4
Comorbidities	79				
Yes	…	41/79 (51.9%)	30/66 (45.5%)	11/13 (84.6%)	**.010**
No	…	38/79 (48.1%)	36/66 (54.5%)	2/13 (15.4%)	
Diabetes	73	8/73 (11.0%)	7/60 (11.7%)	1/13 (7.7%)	>.9
HIV	76	20/76 (26.3%)	13/64 (20.3%)	7/12 (58.3%)	**.011**
AIDS	73	9/73 (12.3%)	7/60 (11.7%)	2/13 (15.4%)	.7
Silicosis	73	1/73 (1.4%)	1/60 (1.7%)	0/13 (0.0%)	>.9
Chronic kidney disease	73	4/73 (5.5%)	2/60 (3.3%)	2/13 (15.4%)	.14
Neoplasm	73	7/73 (9.6%)	4/60 (6.7%)	3/13 (23.1%)	.10
Change of cancer condition	73	4/73 (5.5%)	1/60 (1.7%)	3/13 (23.1%)	**.016**
Total number of comorbidities^b^ Median (Q1, Q3)	79	1.00 (0.00, 2.00)	0.00 (0.00, 1.00)	2.00 (1.00, 2.00)	**.004**
Chest radiograph	59				
Normal	…	3/59 (5.1%)	2/46 (4.3%)	1/13 (7.7%)	.5
Pathological without cavitation	…	39/59 (66.1%)	30/46 (65.2%)	9/13 (69.2%)	>.9
Pathological with cavitation	…	29/59 (49.2%)	25/46 (54.3%)	4/13 (30.8%)	.13
Follow-up treatment	…				
Lost to follow up	…	34/41 (82.9%)	30/36 (83.3%)	4/5 (80.0%)	>.9
Treatment adherence	…	26/38 (68.4%)	21/33 (63.6%)	5/5 (100.0%)	.2
Death	…	11/53 (20.8%)	9/46 (19.6%)	2/7 (28.6%)	.6
Cluster membership	79				
First infection	…	51/79 (64.6%)	45/66 (68.2%)	6/13 (46.2%)	.2
Last infection	…	55/79 (69.6%)	45/66 (68.2%)	10/13 (76.9%)	.7
Change of cluster membership	…	6/79 (7.6%)	0/66 (0.0%)	6/13 (46.2%)	**<.001**

^a^Social class according to the Spanish Society of Epidemiology Report (Supplementary Table 1).

^b^Total number of comorbidities includes other chronic diseases such as chronic obstructive pulmonary disease, hepatitis, syphilis, chronic alcoholic pancreatitis, etc. Significant values are marked in bold.

**Table 2.** Antituberculous Drug Susceptibility Patterns in Recurrent Cases

	Reactivations (n = 66)		Reinfections (n = 13)	
Antituberculous drug	First episode	Subsequent episode	First episode	Subsequent episode
**Rifampicin**	65 susceptible	61 susceptible	All susceptible	All susceptible
		4 resistant		
	1 resistant	1 resistant		
**Isoniazid**	58 susceptible	51 susceptible		
		7 resistant		
	8 resistant	1 susceptible		
		7 resistant		
**Ethambutol**	All susceptible	All susceptible		
**Streptomycin**	61 susceptible	59 susceptible		
		2 resistant		
	4 resistant	1 susceptible		
		3 resistant		
	1 unknown	1 unknown		

### Molecular Characteristics

**Figure 3.**
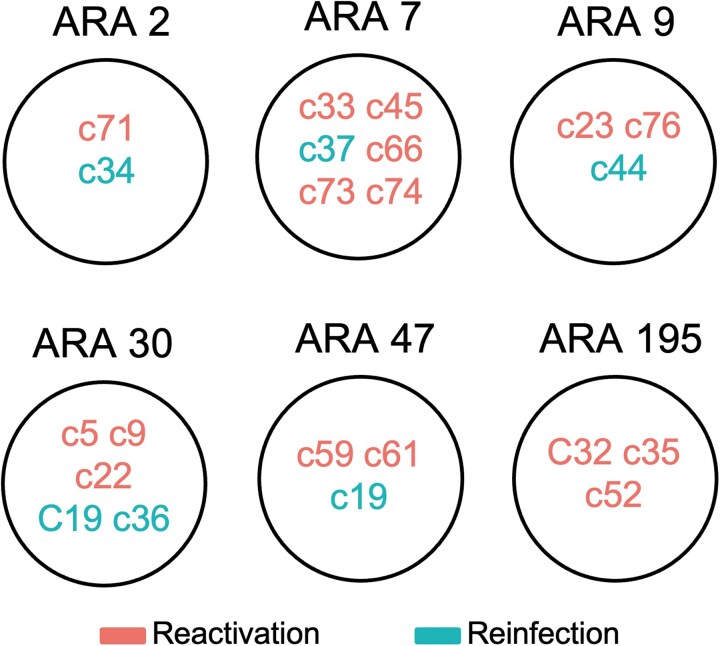
Recurrence cases aggregated into the largest clusters within our population. Cases: c34, c36, c37, and c44 represent isolates from the second episode of reinfection cases. For reinfection c19, the initial isolate was identified in ARA 30, whereas the subsequent isolate grouped with ARA 47.

Almost all *M tuberculosis* strains identified belonged to L4 (98.6%), with only 1 reinfection case caused by a strain identified as *M africanum* (L6). Regarding strain clustering, 46.2% (6/13) of the strains responsible for the first episode of reinfection were part of a previously identified cluster, whereas this proportion increased to 76.9% (10/13) during the second reinfection episode. In reactivation cases, 68.2% (45/66) of the strains were linked to existing community clusters. However, these differences were not statistically significant. Twenty-two recurrent cases were grouped into 6 major clusters, each containing more than 9 cases that had circulated previously. These clusters are detailed in Figure 3. Of the 13 patients with exogenous reinfection, 8 were native and 5 were foreign-born (c3, c8, c12, c14, and c37). A more detailed analysis showed that c3, originally from the Democratic Republic of the Congo, was initially infected with an orphan strain, whereas the reinfecting strain belonged to a community cluster. Cases c8 and c14, from Equatorial Guinea and Romania, respectively, were infected by orphan strains in both episodes. In c14, classification was initially unclear because of the similarity in the RFLP patterns of both isolates and differences in spoligotyping profiles and WGS confirmed the presence of 2 distinct strains, ruling out endogenous reactivation [15]. Case c12, from Equatorial Guinea, was considered the index case of a new cluster based on the timeline of strain appearance in the community. In its second episode, this patient was infected with a previously unreported L6 strain. Case c37 coming from Republic of Cabo Verde, was infected by 3 different strains, all of them belonging to active clusters in the population.

## DISCUSSION

This 30-year molecular epidemiology study in Aragón, Spain, a region with low TB incidence, demonstrates that TB recurrence is a relatively infrequent phenomenon, affecting only 2.30% of patients with genotyped isolates. Endogenous reactivation accounted for the vast majority of recurrent cases (1.93%), whereas exogenous reinfection was less common (0.42%). These findings are consistent with those from other low-incidence settings in Europe and reinforce the notion that reactivation remains the predominant mechanism of recurrence where TB transmission is under control [9]. According to WHO, recurrent TB constitutes approximately 7% of incident cases [5]. Our findings, which reveal a recurrence rate below that threshold, align with other Spanish studies, 1 conducted over 13 years in Getafe (Madrid) and another over 6 years in Gran Canaria, which reported recurrence rates of 1.24% and 2.32%, respectively [23, 24].

A systematic review by Vega et al. of more than 48 molecular studies confirmed that endogenous reactivation dominates in low-incidence countries [9]. Most studies had limitations like short follow-ups, selective cohorts, or lacked population-based design. In contrast, our study provides long-term, comprehensive data from a stable population with universal healthcare. Reported recurrence rates varied widely (0.40%–63.27%) because of heterogeneity and different goals, including treatment comparisons or specific populations (eg, extensively drug-resistant TB, HIV, cancer). Few studies showed higher reinfection rates, mainly in high-incidence areas like South Africa and China [25, 26]. Vega et al. noted that increased TB incidence correlates with higher reinfection, reducing reactivation dominance and identified HIV as a moderate risk factor for reinfection [9, 27]. Our study highlights that mobility, HIV, cancer status, and multimorbidity are key but often underrepresented risk factors. A high number of the cases presented at least a comorbidity (84.8%), suggesting a poor health status.

The comparison between reactivation and reinfection found that most of the cases were male (81.8% vs 69.2%) and had a median age of 39.0 versus 36.0 years at the first episode and 42.0 versus 46.5 years at the last episode. Most were smokers (84.3% vs 75.0%) and reported habitual alcohol use (79.2% vs 81.8%). These features reflect a typical profile and align with known risk factors: male sex [28], age 30–59 years [11], smoking [10], and alcohol use [29]. More than half of reinfection cases (66.7%) changed primary healthcare areas, compared to 15.4% of reactivation cases, suggesting that residential instability may be a risk factor for reinfection, likely linked to socioeconomic or immigrant status, frequent relocations, unstable jobs, and poor housing, factors increasing exposure after treatment. Interestingly, treatment adherence, often associated with TB recurrence [12], did not differ significantly between groups in our study. However, drug resistance, notably to isoniazid and rifampicin, was observed exclusively among reactivation cases, reinforcing its link with subclinical persistence and treatment failure [30].

Classic risk factors for recurrence include diabetes mellitus, HIV infection, and chronic pulmonary disease [10, 11, 31]. Diabetes mellitus and cavitary disease has been suggested as an individual risk factor for reactivation [7, 32], whereas HIV infection has been associated with reinfection [31, 33]. In our study, HIV infection was significantly associated with reinfection. Additionally, individuals with reinfection had a higher average number of comorbidities compared to those with reactivation, suggesting a poorer general health status and greater clinical complexity in the reinfection group. These host-related vulnerabilities also support the dynamic model of TB pathogenesis proposed by Behr et al., which moves beyond binary definitions of latent versus active TB [16]. Their work highlights that the first 2 years after infection or treatment represent the highest risk period for reactivation. Our findings confirm this: endogenous reactivations occurred mostly within 2 years, whereas reinfections appeared much later (median 7 years), likely reflecting new exposure. These temporal dynamics underscore the importance of sustained clinical follow-up, particularly during the early posttreatment period. This finding supports the theory that reactivation risk decreases over time, occurring primarily within the first year posttreatment, whereas reinfections tend to occur later [16]. Therefore, studies with shorter follow-up periods may underestimate reinfection rates. Moreover, the most reinfections involved strains were already circulating in the community, indicating persistent but low-level transmission. Most TB cases were caused by *M tuberculosis* L4; only 1 reinfection was attributed to L6 in a foreign-born patient, likely imported from their country of origin. Although the Beijing genotype (L2) has historically been associated with recurrence, especially reactivation [13], it was not identified in any of our recurrent TB cases. In contrast with findings in Gran Canaria, where L2 was frequently isolated in recurrent cases [24]. That all reinfections with orphan strains involved foreign-born individuals, including 1 L6 case, is consistent with prior findings linking migration and travel from high-incidence countries to reinfection risk [34].

According to the hypothesis that reinfections increase with rising TB incidence [9], one would expect reinfecting strains to be more frequently found in transmission clusters, as patients are more likely to be reinfected with circulating strains. Supporting this theory, 10 of the 13 reinfection cases in our study belonged to clusters, indicating ongoing community transmission. The 3 unclustered strains from foreign-born patients likely resulted from travel-related reinfection, explaining the lack of local clustering. Some large clusters included both reactivation and reinfection cases, suggesting persistent strains capable of causing both. This raises the possibility that some reinfections with genetically identical strains are misclassified as reactivations, as IS*6110*-RFLP has limited resolution. Higher resolution methods like WGS are needed for better discrimination. Our study with WGS confirmed genome similarity in reactivation cases [15].

Our results also contribute to the debate on vaccine trial design. The phase 2b trial by Borges et al., which evaluated the H56:IC31 vaccine in previously treated patients, demonstrated modest efficacy in preventing recurrence [17]. However, it also exposed the limitations of using recurrence as a vaccine endpoint in settings where such events are infrequent and mechanistically heterogeneous. In our cohort, the rarity of recurrence and the long latency of reinfections further limit the feasibility of recurrence-based endpoints. These data support the global shift, reflected in WHO's Preferred Product Characteristics, toward prevention of disease as the preferred primary endpoint for TB vaccine trials.

Finally, the limitations of our study are inherent to its retrospective nature. First, we lost TB cases without a positive culture as our starting point was the genotyped *M tuberculosis* strains. The lack of homogeneity in medical records over time led to missing data in key variables. This issue may have been worsened by patient transfers between health facilities, potentially resulting in underestimation of recurrence. Because of the lack of individual follow-up data, incidence rates expressed in person-time could not be estimated. Additionally, it was not possible to verify treatment adherence in all patients, which is crucial for confirming that recurrent cases were not due to incomplete treatment or loss to follow up. Furthermore, some cases may have been excluded due to insufficient or poor-quality samples for genomic analysis, limiting full coverage. Regarding the molecular technique used, RFLP, despite its wide application, has limitations in differentiating strains with similar genotypic patterns. However, most reactivation cases were analyzed using WGS in a previous study by our group [15], significantly reducing the risk of false positives in reactivation classification and strengthening the validity of our findings.

Our findings highlight the importance of long-term molecular surveillance. Tuberculosis recurrence, though rare, mostly results from reactivation within 2 years, emphasizing targeted clinical monitoring then. Detection of late reinfections, especially in vulnerable or foreign-born individuals, underscores the need to address social factors, improve care continuity, and maintain genomic surveillance to spot transmission changes. Our study supports current global vaccine and recurrence prevention strategies, providing a benchmark for future interventions. Even in well-resourced settings, TB recurrence remains a concern, particularly for patients with HIV, neoplasm, comorbidities, or high mobility. Molecular epidemiology is vital for understanding recurrence mechanisms and transmission. Ongoing vigilance and targeted prevention are essential to advancing TB control and preventing recurrence in vulnerable groups.

## Supplementary Material

ofag079_Supplementary_Data

## Supplementary Data

Supplementary materials are available at *Open Forum Infectious Diseases* online. Consisting of data provided by the authors to benefit the reader, the posted materials are not copyedited and are the sole responsibility of the authors, so questions or comments should be addressed to the corresponding author.
