# Supplementary material for: Factors Involved in Tuberculosis Recurrence in a Low-incidence Setting; Reactivation Predominates Over Reinfection in a 30-year Surveillance Study
Source: Open Forum Infect Dis. 2026 Feb 18;13(3):ofag079. doi: 10.1093/ofid/ofag079 (PMC12989741; doi:10.1093/ofid/ofag079)
Supplement: ofag079_Supplementary_Data [file ofag079_supplementary_data.docx]

**Supplementary Table S1**. Social class according to the Spanish Society of Epidemiology (SEE) Report

| **I** | Senior managers in Public Administration and in companies with 10 or more employees.  Professions requiring second- and third-cycle university degrees. |
| --- | --- |
| **II** | Managers of companies with fewer than 10 employees.  Professions requiring a first-cycle university degree.  Technicians and associate professionals.  Artists and athletes. |
| **IIIa** | Administrative employees and professionals supporting administrative and financial management.  Personal service and security workers. |
| **IIIb** | Self-employed workers. |
| **IIIc** | Supervisors of manual workers. |
| **IVa** | Skilled manual workers. |
| **IVb** | Semi-skilled manual workers. |
| **V** | Unskilled workers. |
